# Second Harmonic Generation for Moisture Monitoring in Dimethoxyethane at a Gold-Solvent Interface Using Plasmonic Structures

**DOI:** 10.3390/nano9121788

**Published:** 2019-12-16

**Authors:** Hannah Aharon, Omer Shavit, Matan Galanty, Adi Salomon

**Affiliations:** Department of Chemistry, BINA Nano Center for Advanced Materials, Bar-Ilan University, 5290002 Ramat-Gan, Israel; hannaharon@gmail.com (H.A.); shavit.omer.gcr@gmail.com (O.S.); matangalanty@gmail.com (M.G.)

**Keywords:** second harmonic generation (SHG), plasmonic enhancement, second-order nonlinear susceptibility, battery

## Abstract

Second harmonic generation (SHG) is forbidden from most bulk metals because metals are characterized by centrosymmetric symmetry. Adsorption or desorption of molecules at the metal interface can break the symmetry and lead to SHG responses. Yet, the response is relatively low, and minute changes occurring at the interface, especially at solid/liquid interfaces, like in battery electrodes are difficult to assess. Herein, we use a plasmonic structure milled in a gold electrode to increase the overall SHG signal from the interface and gain information about small changes occurring at the interface. Using a specific homebuilt cell, we monitor changes at the liquid/electrode interface. Specifically, traces of water in dimethoxyethane (DME) have been detected following changes in the SHG responses from the plasmonic structures. We propose that by plasmonic structures this technique can be used for assessing minute changes occurring at solid/liquid interfaces such as battery electrodes.

## 1. Introduction

The characterization of interface surfaces is the first step towards improving the performance of electronic devices while being essential for understanding chemical transformations occurring on (electrode) surfaces. For example, a characterization of solid-liquid interfaces is very important for the field of batteries and can lead to improvements that are otherwise not feasible [1,2,3,4,5]. Many of the surface-characterization tools are limited to samples situated in a vacuum and therefore in-situ and in-operando experiments are less applicable to these techniques, although much improvement has been seen in recent years [6,7]. Optical techniques, on the other hand, are more flexible and less demanding in terms of sample handling, but usually suffer from lack of surface sensitivity with signals from the surface often buried in the bulk. Two-photon microscopy is a versatile optical tool for probing surfaces and interfaces in material science, opening vast perspectives in clinical cancer research and for neuroscience imaging [8,9,10]. This includes two-photon excitation fluorescence (TPEF), second harmonic generation (SHG) and sum frequency generation (SFG). TPEF is an incoherent process whereby two photons are simultaneously absorbed to produce a single fluorescence photon after some delay and with energy losses. In contrast, SHG and SFG are coherent processes with no *absorption* of photons by the sample and no energy losses. In general, two photons at the same ω frequency for SHG or at different ones ($\omega_{1}\neq \omega_{2}$) for SFG, combine to generate a single photon at the double frequency 2ω or the sum frequency $\omega_{3}=\omega_{1}+\omega_{2}$ respectively [11]. Such configurations can lead to spectroscopic information that is not accessible by way of the classical one-photon linear spectroscopy tools that are governed by restricting dipolar selection rules. SFG thus, is an intrinsically surface-selective vibrational spectroscopy method [12,13,14,15,16]. A specific and simplified form of this is the use of two photons with the same fundamental frequency ω combined to generate a single photon of frequency 2ω, as in SHG [11].

Optical nonlinear effects are inherently weak and they are dominated by photon-photon interactions, processes enabled by material properties. Such effects are accounted for by perturbative alteration of the usual linear light-matter interaction models. According to the electric-dipole approximation, a medium deprived of inversion symmetry is required at both the atomic or molecular scale and at the crystalline or supra-molecular organization levels. Accordingly, breaking the symmetry at the interface leads to an additional specific surface-sensitive SHG response which can be used to probe phenomena occurring at the interface [11,14,17,18,19,20,21,22].

SHG from metallic surfaces is normally dominated by the nonlinear polarizability of the free and bound metal electrons at the interface. The motion of the oscillating electrons under the fundamental electric field gives rise to a macroscopic time-dependent polarization [11]. Therefore, the SHG response should be changed upon molecular adsorption or any other chemical process occurring on the metallic surface, as a result of modification of the polarizability at the interface [9,23].

The change in the SHG response is described by the surface nonlinear susceptibility, $\chi^{\left(2\right)}$, which is directly related to the symmetry of the measured object and can be determined from polarization-dependent measurements [24] (as from SHG rotational anisotropy measurements), and the magnitude of the SHG responses, resulting in specific organization or resonance dependent angular plots [9]. Thus, $\chi^{\left(2\right)}$ is dependent on the shape of the electron-density profile at the surface.

When a chemical event occurs at the interface, it induces a change in the electronic distribution further leading to a modification of the polarizability and thus of the $\chi^{\left(2\right)}$ tensor. Such a change can be decomposed into various contributions according to the following expression [9,25]:

(1) $$\chi^{\prime \left(2\right)}=\chi^{\left(2\right)}+\chi_{A}^{\left(2\right)}+\Delta \chi_{I}^{\left(2\right)},$$

where $\chi^{\prime \left(2\right)}$ stands for the overall modified surface susceptibility.

The change of the susceptibility occurs due to the adsorbate itself, for example due to the attachment or proximity of some molecular species $\chi_{A}^{\left(2\right)}$, and the interaction between the adsorbate and the surface $\Delta \chi_{I}^{\left(2\right)}$.

The motivation of the following study is to maximize $\Delta \chi_{I}^{\left(2\right)}$ in order to increase the overall SHG response to gain as much information as possible as to the molecules/solution at the interface under investigation.

For this purpose, we used triangular plasmonic cavities milled in Au thin film which have been reported to give rise to significant and measurable SHG responses [26,27]. If close enough, those cavities can undergo coupling and the SHG response is dramatically enhanced, as reported earlier [26,27,28,29,30,31]. The interaction between the adsorbate and the plasmonic cavities can alter the coupling between the cavities and the overall susceptibility at the interface, allowing us to measure small events occurring on the metallic surface.

Metallic nanostructures are well-known to enhance nonlinear responses [32,33,34,35,36], which is ascribed to the excitation of surface plasmons (SP’s). SP’s are coherent oscillations of the metal-free electrons which, under appropriate conditions such as sub-wavelength apertures may couple to the electromagnetic (EM) field in their vicinity and lead to a confined mode that is either localized or allowed to propagate along with the interface [35,37,38]. The geometrical parameters of the metallic nanostructures (particles or cavities) determine the electric-field enhancement and confinement at specific frequencies. At specific optical frequencies, these collective oscillations produce large polarizabilities which reinforce the local EM field and enhance both the linear and nonlinear optical response of the system [35]. Although enhanced SHG was observed from silver and gold island films already back in 1981 [39], the field has been revived by the development of unique fabrication and testing toolbox of nanotechnologies allowing to pattern surfaces with nanometer precision, turning it into an active ongoing research domain [26,40,41,42,43,44,45,46,47,48]. Properly tuning the thickness of gold layers in conjugation with nano-particles, such as ZnO, allows the enhancement of the optical properties of these particles and can thus also enhance the nonlinear responses [49].

Herein we focus on gold electrode/Dimethoxyethane (DME) interface because DME has been explored as a solvent in the use of rechargeable magnesium batteries and is of great interest to the electrochemistry community [50,51]. More specifically, it has been noted that in the case of electrochemical cells, dehydration plays an extremely important role, as small amounts of water contamination can change the orientation and organization of DME molecules at the electrode vicinity [52], and interact with other solvents and materials used, thus leading to degradation in their performance. Besides the fundamental investigation itself, the ability to monitor such contamination is therefore of great practical prominence.

## 2. Materials and Methods

Sample preparation—Fused silica substrates were cleaned using diluted Hellmanex™ III (Hellma GmbH, Müllheim, Germany) (1:100) in a sonication bath for 20 min at 40 °C. Substrates were then washed with double distilled water (DDW) and ethanol and dried using a nitrogen gun (99.999%).

**Figure 1:**
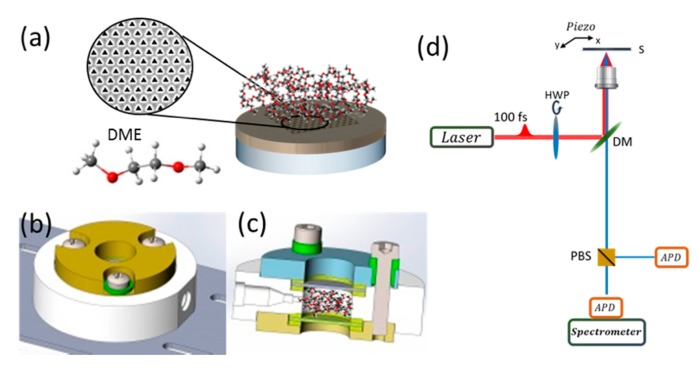
General description of the setup used. Schematic illustration of electrode-molecule system: (**a**) electrode comprising a glass substrate with a 200 nm thick layer of Gold in which a hexagonal array of equilateral triangles has been milled. Solvent molecules are shown on top of the electrode. SEM image of the array is shown in the inset. Bottom left: illustration of the solvent molecule DME (dimethoxyethane), (**b**) Top view of the cell in which substrate and solvent are placed (**c**) Side slice view of the cell: chamber height—5 mm; total cell height 15.9 mm. The main body is made of PEEK (polyether ether ketone), the top and bottom parts are made of stainless-steal (**d**) Schematic illustration of the SHG microscopy setup. The fundamental excitation polarization is rotated with a half-wave plate (HWP). The beam is at 90 degrees with respect to the sample plane. A dichroic mirror (DM) is used to direct the light upward to the sample (S), which is mounted on a piezo actuator (Piezo) to allow high accuracy scanning. The SH beam leaving the microscope can then be re-directed using a flip mirror (FM) towards a spectrometer equipped with an electron multiplier charge-coupled device (EMCCD), or onto a polarized beam splitter (PBS) coupled to two avalanche photodiodes (APDs).

The clean substrates were coated by 5 nm of chromium and 200 nm of gold using E-beam evaporation in high vacuum. Chromium was used as an adhesion layer for gold. The adhesion layer was important in this case to stop the gold from pealing and falling into the solvent. Gold was used because it is relatively stable, doesn’t oxidize or react with DME. Arrays of equilateral triangular cavities with a side-length of 200 nm and a spatial distance of 400 nm centre-to-centre were fabricated by focused ion beam (FIB, FEI, Helios Nano Lab 600i) (see Figure 1a). The array was used for SHG enhancement. Figure 1a shows a schematic description of the sample.

Cell Setup—A cell was specifically designed to allow measurements with solvents in an isolated environment (see Figure 1b,c). The cell was fabricated from stainless steel and polyether ether ketone (PEEK). The main body was made of PEEK for chemical stability. This part was sandwiched by stainless steel parts to allow electric conductivity when needed. Gaskets made of Kalres^®^ were placed between the PEEK, stainless steel and glass slides. One of the glass slides has the fabricated sample on it and is placed facing into the inside of the cell such that when the cell is filled with solvent the sample is immersed in it. All parts are held together by stainless steel screws which are isolated from the top stainless-steel body by PEEK parts. In this configuration, the screw heads and the top stainless-steel part can be used as electric contacts to opposite sides of the cell (the screws are directly connected to the bottom stainless steel part of the cell and isolated from the top part). The main part made of PEEK has an inlet and an outlet to allow the flow of fluid or gas. Chamber volume is 0.5 mL. A detailed figure of the cell can be found in Figure S1.

The studied sample was placed in the cell. Then, the cell was tightly closed and filled with dry DME (Merck) in Ar environment inside a glovebox. After the SHG measurements, the dry DME in the cell was exposed to ambient conditions in the lab (60% humidity) for one minute, allowing absorption of ppm amounts of $H_{2}O$. This was assessed by Karl-Fischer measurements. There was a 40-fold increase of $H_{2}O$ molecules in the sample after exposure than before, i.e., an increase from 2.5 ppm to 100 ppm, respectively. After this exposure, the cell was tightly sealed so no other changes could occur and no additional water could be adsorbed.

Figure 1 shows a scheme of the experimental setup. The gold coated glass substrate on which the plasmonic array is milled is immersed in DME, Figure 1a with the inset showing an example of the plasmonic structure scanned and the molecular structure of the solvent. A top (Figure 1b) and slice (Figure 1c) view of the chamber used with solvent molecules emphasizing the size and position of the area being filled. A scheme of the two-photon microscope setup used is shown in Figure 1d with the important optical elements elaborated. We note that such a cell can be used for future in-operando experiments.

Scanning SHG—The generation of SH signal was performed using a mode-locked tunable (690 nm–1040 nm) Ti:Sapphire laser (Spectra-Physics Mai-Tai HP, 100 fs, 80 MHz). The laser showed high output power stability over long periods of time and measurements.

Essential components of this setup are (a) an attenuation unit: an achromatic half-wave plate (HWP, Thorlabs INC, Newton, NJ, USA) coupled to a Glan-Taylor polarizing beam splitter (GP, Thorlabs), allowing control over the output laser intensity, (b) another achromatic half-wave plate (HWP, Thorlabs), mounted on a motorized rotation stage (Thorlabs), and (c) a polarizing beam splitter (PBS, Thorlabs) in the detection path. (b) and (c) allow for measurement of the incident polarization dependence of the emitted signal for different structures and solvents. Precise rotation of the HWP turns the incident polarization angle, while the PBS works like an analyzer, which separates the emitted SH signal into two different detection channels with orthogonal polarization components. Detection was performed by two single-photon avalanched photodiodes (APD, Blue Count, Laser Components) coupled to two optical fibres (OF). The dark noise of the APDs was in the range of 0–100 cps, with a dead time of ~43 nm.

The fundamental laser light was directed into an inverted microscope (IX73 series, Olympus, Tokyo, Japan) with chosen polarization and radiation power. At the microscope entrance, the beam is reflected by a dichroic mirror (DM, Chroma), with cutting wavelength at 700 nm, fixed at 45°.

Since samples used were opaque and highly reflective, it is safe to say that for all practical purposes, all the generated SH photons were scattered in the backward direction. In order to allow plasmonic excitation and collection of the emitted signal, a long working distance 50× objective lens NA = 0.5 (Olympus) was used. The NA used in such cases is usually high, to obtain a tightly focused excitation volume with high pulsed peak intensity. The collected signal and the effect shown in this study is high and efficient, even though the NA of the objective was low. The DM spectrally separated the fundamental excitation beam from the emitted SHG signal. Additional short-pass and band-pass filters (SPF and BPF, Semrok, Rochester, NY, USA) were placed in the collection path for further spectral filtration of the fundamental beam, two-photon excited fluorescence (TPEF) and other two-photon processes. SH intensity maps were collected by raster scanning the sample surface using a piezo positioning stage (Piezosystem, Jena, Germany XY positioning series). SH emission was verified by measuring the emitted spectrum signal with a spectrograph (Shamrock 303i) equipped with an electron multiplier charge-coupled device (EMCCD) camera (Andor Newton). Though there is some loss of signal using OFs, they are used to reduce background noise effectively.

We first measured the nonlinear responses of the plasmonic array covered with a protective PVA layer, without the liquid cell as shown in Figure S2.

A comparison of the same array in three different environments, showing the change of intensity due to changes in the solvent was performed (see Figure S3). A further comparison of the average pixel intensity from the plasmonic structure of the same array in the different environments and the surface nearby these structures is shown in a histogram (see Figure S4).

The signal-to-noise ratio (SNR) from the structure covered with PVA is much higher compared to that in the solvent for an obvious reason: the PVA layer is of the order of ~150 nm, and the solvent is almost 0.5 cm thick including an additional glass cover. This causes significant scattering of both the fundamental light and the SH emission. Therefore, a much higher fundamental intensity was needed. The polar plot obtained is similar in shape, which is to be expected when looking at arrays with the same parameters, namely, structures with three-fold symmetry [28,53]. Changes in the polar plot arise from changes in the coupling of the triangles in the array caused by the solvent, an effect to be addressed in subsequent investigations, whereas we are presently focusing on the role of the structure towards SHG enhancement.

Quadratic Dependence—The SHG signal was collected at different fundamental laser power and plotted in log-log scale, to evidence the quadratic dependence of the SHG signal with respect to the laser power, as expected from a quadratic phenomenon (Figure S5).

SHG Spectra—SHG spectra were obtained as explained above. The Spectra were collected for various fundamental wavelengths, from 840–1000 nm with 20 nm steps (Figure S6). The SHG signal was clearly observed at the expected harmonic wavelengths, i.e., 2 ω. In addition, other nonlinear processes such as fluorescence were also observed at higher wavelengths with a clear cutoff at 680 nm due to a low-pass filter. Additional filters were used to eliminate the fluorescence and fundamental laser residues.

Angle-Resolved SHG—Polarization resolved SHG were obtained by rotating a half-wavelength plate ($\frac{\lambda }{2})$ through which the fundamental beam was directed causing a rotation of the input polarization by 8° steps. Acquisition time was set to 500 ms. The two branches of the PBS correspond to two orthogonal polarizations of the SHG signal emitted from the sample (along the *x* and *y* axes respectively corresponding to s- and p-polarization).

Polar dependent plots were acquired several times for each array in different conditions to check the consistency and stability of the system. Each measurement was performed at a slightly different location of a given array with a spacing of the order of 0.5–1 μm. Measurements have also been repeated on different samples, see Figure S7.

Linear spectra of the sample with PVA and DME were collected to ensure off-resonance response for both cases, thus eliminating any resonance enhancement that may occur from a change in refractive index, Figure S8.

SEM—To check whether the sample degrades with time, i.e., started to peel or encountered any changes to the surface, SEM images were obtained pre and post SHG measurements. These images show that no such changes were made to the surface and the metallic film remained intact throughout measurements (see Figure S9).

## 3. Results and Discussion

**Figure 2:**
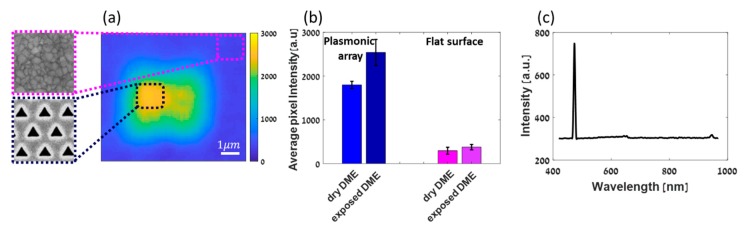
(**a**) SHG intensity map of an array in DME after exposure to ambient conditions. Samples of areas scanned are enlarged (not to scale). (**b**) Average pixel intensity from three different arrays of the same parameters (shape size and periodicity) in two different environments—dry DME and DME after being exposed to ambient conditions, compared to the average pixel intensity from the flat surface around the arrays calculated with an area of approximately 1×1 μm. Areas used for calculations are marked by dotted lines—black for the plasmonic array and pink for the flat surface. (**c**) Spectrum obtained from the sample, showing the sharp SHG signal. Filters were removed, showing that very little fluorescence and fundamental residues are left at ~650 and 940 nm, respectively.

Figure 2 shows the SHG scanning of a hexagonal triangular plasmonic array milled in Au, covered with 5 mm (or 0.5 mL) of DME solution (see Figure 1). The relatively high SHG response with respect to the surface originates from the plasmonic structure and it is at least 3 times higher compared to a smooth Au film. Additionally, one can notice that the SHG response from the plasmonic structure is drawn inwards towards the centre and decays towards the edges. This is due to plasmonic coupling between the triangular holes which are 400 nm apart from each other, in agreement with previously reported studies [28]. At about one micron from the plasmonic structure the signal has decayed completely and becomes very low arising only from the Au surface and its interaction with DME molecules (see Figures S3 and S4).

Next, we compared the SHG responses of the cell with dry DME to that of the cell after a short time exposure to ambient conditions, allowing absorption of ppm amounts of water as assessed by Karl Fischer measurements. A histogram of the average responses per micron square is shown in Figure 2b. About 50% change in the SHG intensity from the plasmonic structure before and after exposure is observed, unlike the case of the non-structured gold film, in which differences in the SHG responses are within the standard deviation. Those measurements have been repeated several times also with a larger amount of water to assure the directionality of the polarizability change (see Figures S3 and S4).

The relatively large difference of SHG responses emphasizes the sensitivity of the method being presented here.

To ensure that indeed SHG was being collected rather than other fluorescent effects or fundamental light, a spectrum was collected showing the frequency doubling at the appropriate wavelength (Figure 2c). Residues of two-photon fluorescence and fundamental light can be observed at 650 nm and 940 nm, respectively when two filters are removed before collecting the spectrum.

**Figure 3:**
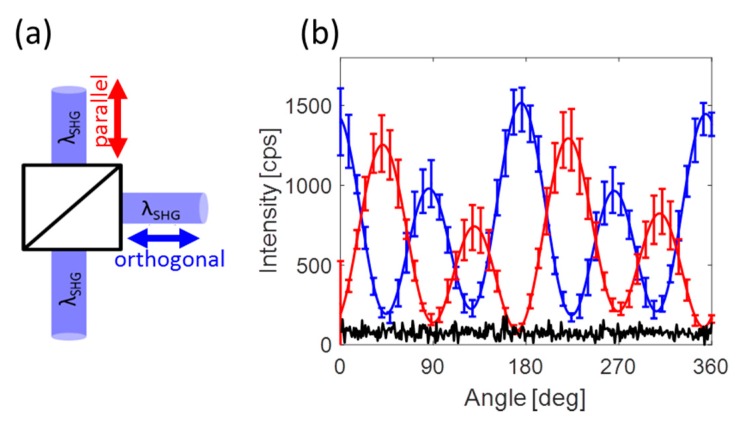
Angle resolved SHG measurements (**a**) The emitted SHG signal $\lambda_{\mathrm{SHG}}$ is directed into a polarized beam splitter (PBS) and is split into two orthogonal polarized emission. (**b**) An average of several measurements with error-bars exemplifying the consistency and repeatability of the system and the measurements performed. The signal was acquired from several locations on the hexagonal hole array. Red and blue plots correspond respectively to two orthogonal output polarizations as is indicated in (**a**). The black line represents the noise level of the detection equipment. Specifically, the measurements were collected from a plasmonic substrate covered by a 5 mm thick solution of dry DME in our customized cell. The error bars and the solid lines correspond to the experimental data and fit, respectively.

To be sure of the consistency and reliability of our system, we ran angle resolved SHG measurements (polar plots) at different locations on the plasmonic hole array. Those measurements have been repeated for several arrays and for each solvent environment. Figure 3b shows an average of several polar plots both in Cartesian coordinates with polarizations both parallel (red) and orthogonal (blue) to the symmetry axis displayed. Repeatability of the measurements is ensured even though the emitted photons are collected after scattering over a 5 mm long propagation through the solvent, as indicated by the error-bars. Small changes of about 20% in amplitude are observed. Yet repeated observation typical four-fold signature of octupolar patterns confirm the relevance of our configuration as a proof that the observed changes in the SHG intensities are generated at the interface and are not due to modifications within the bulk of the metallic structures. In addition, the plots are periodic with an angular period of 180° ending-up after a full revolution of the fundamental polarization at the same intensity started from, thus indicating the stability of our system (Figure S7). The dark noise is of the order of 150 cps. The minimum points of the polar plots are of that order, emphasizing the accuracy of the measurement. An elaborated figure with all measurements used for this average can be found in Figure S7. Here, only the average is shown, to simplify the figure.

The signal from the plasmonic structure is overwhelmed by that from the surface. So much so, that the signal at the minimum point of the polar plots is as low as the noise level (100 cps) and is lower than that of the bare surface, ~500 cps. In addition, no octupole polar plots are observed from the bare surface itself as expected.

**Figure 4:**
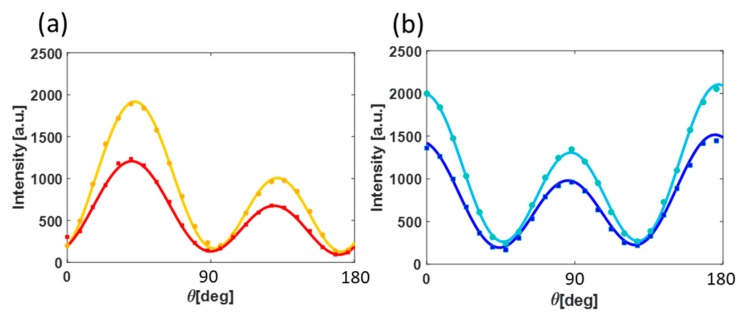
Angle-resolved SHG intensity plots from plasmonic array in different environments and at two orthogonal output polarizations (a + b). Dry DME are in red and blue colours, and DME after exposure to ambient conditions are in orange and light blue colors. (**a**) is for parallel output polarization and (**b**) is for orthogonal output polarization as is indicated in Figure 3a. Points and solid lines are the averaged data and the fit, respectively.

The fact that our polar plots are highly reproducible through 5 mm of solvent encourages us to measure minute changes in the solution using this technique. It is well known that ambient conditions dramatically affect batteries performance. For example, the solubility of electrolytes in DME changes drastically when DME is properly dehydrated because there is a change in the caging of the salt by the DME molecules [50]. Furthermore, following adsorption of water, the solvent (DME in this case) undergoes reorganization, in the bulk as well as at the interface, with clusters formed by water molecules [52]. Figure 4a,b show polar plots obtained respectively from electrode/DME in a dry environment and from an $H_{2}O$ environment. The polar plots were averaged and compared. In agreement with Figure 2b, the amplitude of the SH response after exposure to water is higher as a result of polarizability change at the interface. The SHG responses for metals are dependent on the electron density profile at the interface/surface, which scales nonlinearly with the number of electrons in the system. In this case, it means that the adsorbates which donate electrons to the metallic surface will give rise to enhanced SHG response, whereas molecules (adsorbates) which are withdrawing electrons will decrease the SHG responses.

Indeed, according to the work of Z. S. Nickolov et al., water and DME molecules form clusters. This cluster formation presumably gives rise to changes in the polarizability at the gold surface and thus in the SHG response of the studied plasmonic electrode. We shall note that the index of refraction of DME is 1.377 with slight changes to the third digit when changing the temperature, a similar refraction index to that of water [54]. That is, one can conclude that the changes in the responses of the plasmonic electrode are solely due to reorganization of the solvent at the interface, which results as different polarizability.

For example, it has recently been reported that second harmonic scattering (SHS) was reportedly used to measure the long-range organization of water molecules. Changes in this organization as a function of salt concentration were monitored and reported [55]. Three-dimensional imaging by SHG of water interfacial structure and dynamics in confined space has also recently been reported showing the strength and scope of this technique [56].

## 4. Conclusions

We report herein the use of plasmonic structures as an enhancement for surface chemistry sensing with SHG. Though the change is subtle and cannot be distinguished in the case of a flat surface, a plasmonic array is seen to boost the signal intensity, thus evidencing an interface signal that can be further singled out and analyzed by polarization-resolved acquisitions. Other plasmonic structures such as bow-ties and others which are known to highly boost the EM field can be used for such a study to increase the sensitivity [57,58]. We see it as an important step as the reorganization of the solvent at the interface should lead a different distribution of the electron-density profile at the surface, which in turn should affect the observed polar-plots. Changes in the polar plots following the reorganization of the solvent (DME) at the electrode/liquid interface have been observed by us, yet, the results are too preliminary to draw conclusions at this stage. We think that our proposed methodology is general and can be extended to the sensing of other subtle chemical changes occurring in closed cells, in-situ and possibly also under in-operando conditions through thick layers of liquid. This method could be used for analytical or even bioanalytical purposes [59].

## Supplementary Materials

The following are available online at https://www.mdpi.com/2079-4991/9/12/1788/s1, Figure S1: Our liquid cell with the plasmonic electrode, Figure S2: The plasmonic electrode-SHG scanning, Figure S3 and S4: SHG scanning of the plasmonic electrode in the liquid cell, Figure S5: Quadratic dependence, Figure S6: SHG spectrum, Figure S7: Linear measurement of the plasmonic electrode, Figure S8: Stability of the plasmonic electrode.
