# Supplementary material for: Second Harmonic Generation for Moisture Monitoring in Dimethoxyethane at a Gold-Solvent Interface Using Plasmonic Structures
Source: Nanomaterials (Basel). 2019 Dec 16;9(12):1788. doi: 10.3390/nano9121788 (PMC6955981; doi:10.3390/nano9121788)
Supplement: Supplementary file 1 [file nanomaterials-09-01788-s001.pdf]

nanomaterials

MDPI

Article

Second Harmonic Generation for Moisture Monitoring in Dimethoxyethane at a Gold-Solvent Interface Using Plasmonic Structures

Hannah Aharon, Omer Shavit, Matan Galanty and Adi Salomon *

Department of Chemistry, BINA Nano Center for Advanced Materials, Bar-Ilan University, 5290002, Ramat-Gan, Israel; hannaharon@gmail.com (H.A.); shavit.omer.gcr@gmail.com (O.S.); matangalanty@gmail.com (M.G.)

* Correspondence: adi.salomon@biu.ac.il

Received: 29 October 2019; Accepted: 12 December 2019; Published: date

1. Our liquid cell with the plasmonic electrode

Gold coated glass substrates were placed in a specifically designed cell. This cell is sealed allowing the use of volatile and hygroscopic solvents. It may also be used for *In-situ* and *in-operando* electrochemical measurements. A scheme of the cell is shown in Figure.S1

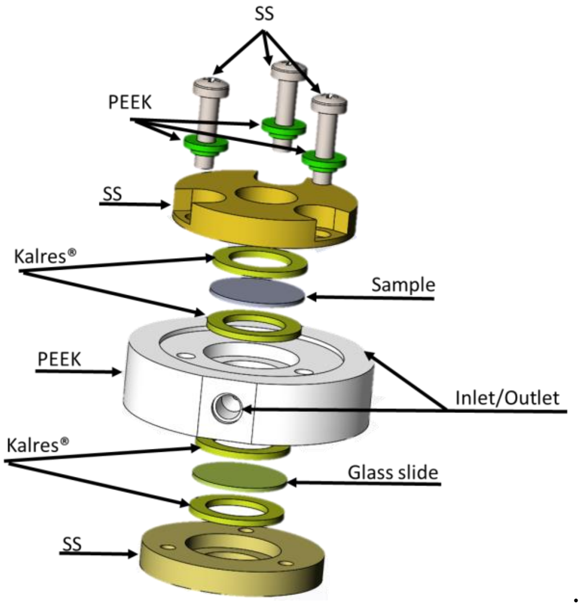

Figure S1. Blown up scheme of cell used showing the different elements used and their chemical components.

2. The plasmonic electrode- SHG scanning

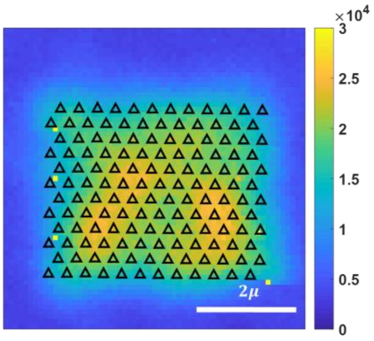

Figure S2. SHG intensity map of the structure used coated by a thin layer (~150nm) of PVA. The triangles are overlaid to guide the eye.

3. SHG scanning of the plasmonic electrode in the liquid cell

A full scan of the structure immersed in dry DME, DME after exposure to ambient conditions and DME with a water concentration of 0.33M were obtained, keeping all other conditions the same. Color-bar is set to be the same for all three scans, emphasizing the increase of intensity with increasing exposure to water. SH response from the surface is not noticeable with the same conditions.

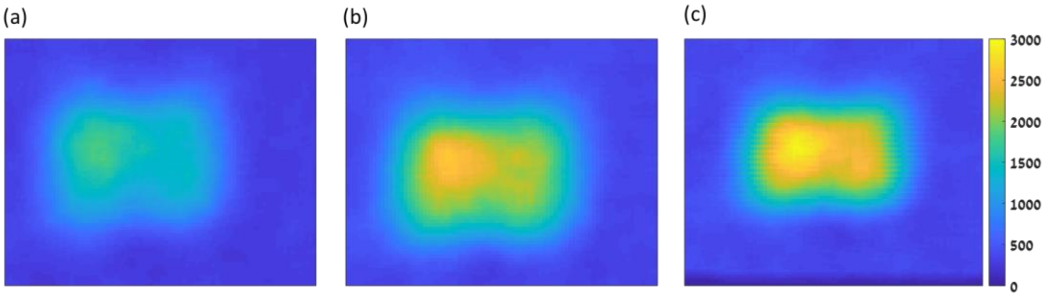

Figure S3. Comparison of structure with different environments. Scaled to the same intensity. A hexagonal array of equilateral triangles with a side length of 200nm and a center-to-center distance of 400nm. Scans were taken in (**a**) DME, (**b**) DME after exposure to ambient conditions, and (**c**) DME/H_2O 0.33M.

After immersing the sample in dry DME and then exposing it to ambient conditions is was then immersed in DME with water at a concentration of ~0.33M. The increase of intensity in this case continues and shows there is a trend to increase from the plasmonic structure. From the surface there is no noticeable change at all.

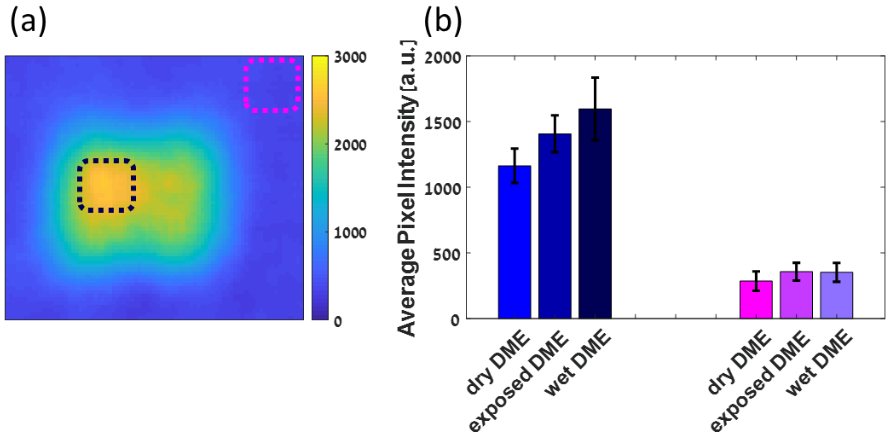

Figure S4. (**a**) SHG intensity map of an array in DME after exposure to ambient conditions. (**b**) Average pixel intensity from three different arrays of the same parameters (shape size and periodicity – **a**, **b** and **c**) in three different environments– dry DME, DME after being exposed to ambient conditions and wet DME (H_2O 0.33M) compared to the average pixel intensity from the flat surface around the arrays calculated from an area of approximately $\mathbf{1 \times 1\mu}$. Areas used for calculations are marked by dotted lines – black for the plasmonic array and pink for the flat surface.

4. Quadratic dependence and SHG spectrum

When measuring SHG, the emission intensity is expected to have a quadratic dependence on the incident power according to

$$log(I_{2\omega}) = log\left[\left(\varepsilon_0 \chi_{eff}{}^{(2)}\right)^2\right] + 2log(I_\omega)$$

Experimentally, the excitation and detection areas are the same as we work in reflection mode. Therefore, the same relationship applies for the power:

$$log(P_{2\omega}) = log\left[\left(\varepsilon_0 \chi_{eff}{}^{(2)}\right)^2\right] + 2log(P_\omega).$$

Therefore, a log-log plot should behave linearly with a slope of 2. The log of the experimental SH intensity is shown as a function of the log of the input laser power. This measurement was performed on a hexagonal array milled in a gold surface as described within a cell and immersed in dry hexane.

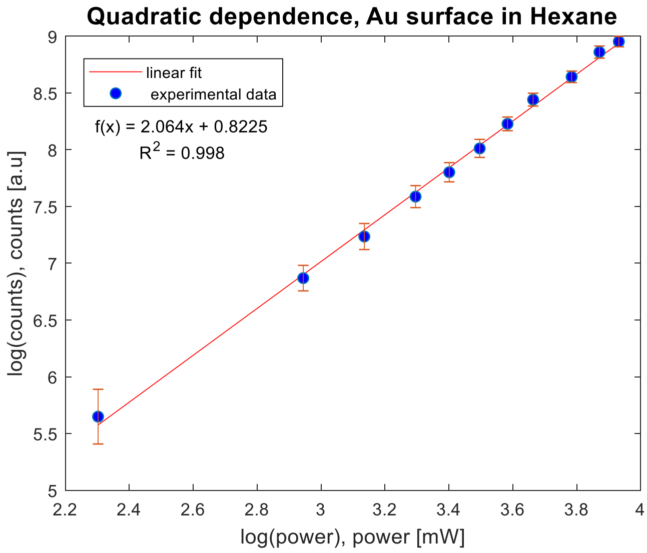

Figure S5. log-log plot of SHG signal verses laser intensity power. The slope is approximately 2 with an R^2 value larger than 0.99, showing that indeed SHG is collected.

SH spectra were obtained at different fundamental wavelengths. We chose a fundamental wavelength of 940nm, at this wavelength there is enough SH response with a minimal amount of fluorescence and fundamental residues, without coming close to a plasmonic mode or being too close to the edge of the lasers range.

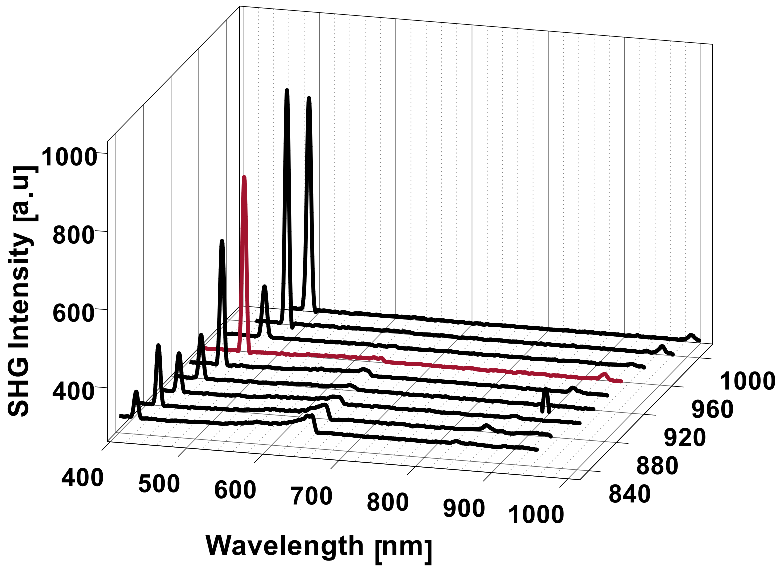

Figure S6. SHG at different fundamental WL varying from 840 to 1000nm with 20nm intervals. Some filters were removed allowing the appearance of some fluorescence and fundamental beam residues. When scanning the SHG intensity plots these filters were placed in the beam path and removed these residues.

5. Angle-Resolved SHG

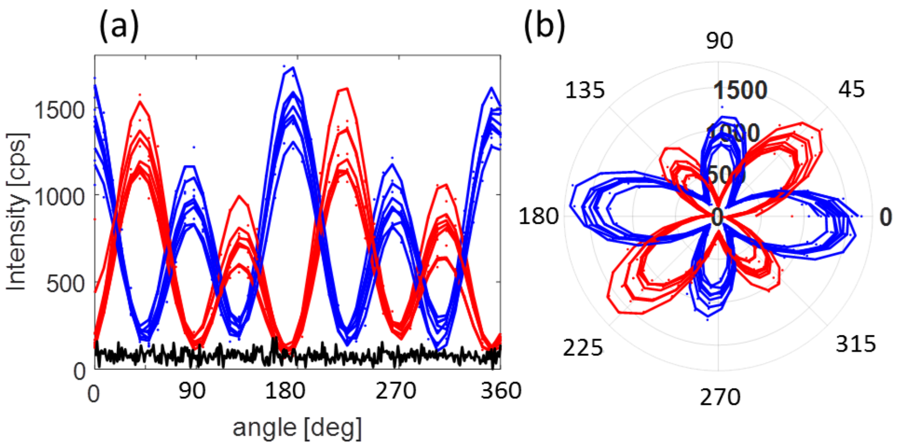

Figure S7. Angle-resolved SHG measurements (**a**) An example of the consistency and repeatability of the system and the measurements performed. The signal was acquired from several locations on the hexagonal hole array. Blue and red plots correspond respectively to parallel and perpendicular output polarizations. The black line represents the noise level of the detection equipment. Specifically, the measurements were collected from a plasmonic substrate covered by a 5mm thick solution of dry DME in our customized cell. The dots and the solid lines correspond to the experimental data and fit, respectively with (**b**) same as (**a**) plotted in polar coordinates.

6. Linear measurement of the plasmonic electrode

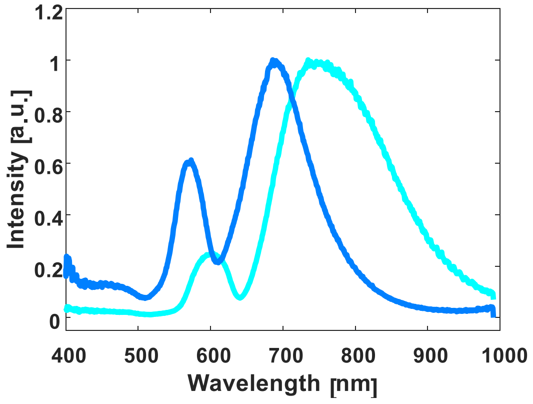

Figure S8. Transmission spectra of the structure with PVA coating – light blue, and immersed in DME – blue line. The plasmon modes are at 748 and 600nm for the PVA coating, and 569 and 690nm for DME. Showing no resonance with the fundamental wavelength used.

Sample was imaged by E-SEM before and after exposure to DME and H_2O to follow the changes to the structure and the surface

7. Stability of the plasmonic electrode

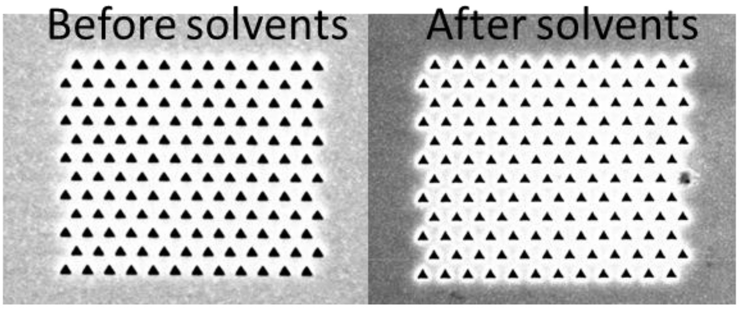

Figure S9. SEM images showing the surface before and after exposure to the solution. There is no corruption or distortion to the structures, nor is there any peeling of the gold film.

Transmission spectra were obtained from the sample in dry DME and with PVA coating. The data was collected using an inverted microscope (Olympus IX83) together with a spectrometer (IsoPlane SCT-320, Princeton Instruments) and a charge-coupled device camera (CCD, PIXS1024b, Princeton Instruments). Samples were illuminated with a non-polarized white light source in bright field. Spectra were collected by a 40x magnification (NA = 0.6) objective. Base line was collected using a clean glass substrate under the same conditions.
